# Incidence of community acquired pneumonia in children aged 2-59 months of age in Uttar Pradesh and Bihar, India, in 2016: An indirect estimation

**DOI:** 10.1371/journal.pone.0214086

**Published:** 2019-03-20

**Authors:** Shally Awasthi, Chandra Mani Pandey, Tuhina Verma, Neha Mishra, Lucknow CAP Group

**Affiliations:** 1 Department of Pediatrics, King George’s Medical University, Lucknow, Uttar Pradesh, India; 2 Department of Biostatistics and Health Informatics, Sanjay Gandhi Postgraduate Institute of Medical Sciences, Lucknow, Uttar Pradesh, India; University of Groningen, University Medical Center Groningen, NETHERLANDS

## Abstract

**Introduction:**

Community Acquired Pneumonia (CAP) is the leading cause of mortality in children younger than five years of age in developing countries, including India. Hence, this prospective study was performed to estimate the incidence of CAP in children (2–59 months)in four districts of Northern India.

**Methods:**

A cross-sectional survey in rural Lucknow was conducted using cluster sampling technique to assess the proportion of CAP cases that were hospitalized in last 12 months (hospitalization fraction). Another prospective study was done to assess number of hospitalized CAP cases in same districts in 2016. For this, a surveillance network of hospitals that admitted children was established. Cases with WHO-defined CAP with less than 14 days of illness were eligible for inclusion. Informed written parental consent was obtained. A mathematical model was developed to estimate the incidence of CAP in each district, taking into account number of cases hospitalized in one year, assuming it to be equal to hospitalization fraction and using Lucknow district as reference, correcting for child-population per hospital for each district. Population census data of 2011 was taken as denominator.

**Results:**

In cross-sectional survey (February to May 2016), 3351 children (2–59 months) from 240 villages were included. Of these 24.58% (824/3351) children suffered from CAP in last 12 months and out of these 4% (33/824) children were hospitalized. Computed incidence of CAP per 1000 child-year for Lucknow was 86.50 (95%CI: 85.72–87.29); Etawah 177.01(95%CI: 175.44–178.58); Patna 207.78 (95%CI: 207.20–208.37) and Darbhanga 221.18 (95%CI: 220.40–221.97). Infants (2–11 months)had almost five to ten times higher incidence of CAP than those in 12–59 months age category.

**Conclusions:**

Incidence of CAP in Uttar Pradesh and Bihar is high, being much higher in infants. Hence there is an urgent need for introduction of preventive strategies, improving health seeking behavior and quality of care for CAP.

## Introduction

Community acquired pneumonia (CAP) is the leading cause of mortality of under-five children in developing countries, including India. Annually there are 151.8 million new cases of CAP [1]. Among these 8.7% (13.1 million) cases are severe enough to require hospitalization [1].Based on burden of CAP, India is among the top five countries and has over 23%of the global cases [2].

World Health Organization (WHO) has developed simple guidelines for the identification of CAP by community health workers [3]. According to these guidelines, CAP is defined as presence of fast breathing above age-specific cutoff. The cutoff for infants less than 2 months is 60 more breaths per minute (bpm), for 2–11 months of age 50 or more bpm and 12–59 months of age is 40or more bpm. In addition, WHO has defined severe pneumonia as CAP with presence of certain danger signs such as not able to drink, persistent vomiting, convulsions, lethargy or unconsciousness, stridor in a calm child or severe malnutrition. Children with fast breathing with or without chest in-drawing are classified as “pneumonia” and children withpneumonia and with any danger signs are classified as“severe pneumonia” [3].

CAP could be of viral or bacterial etiology. Pediatric bacterial pneumonia is predominantly caused by *Streptococcus pneumoniae* (SP) and *Haemophilusinfluenzae type B*(HiB)[4]. In 1990, the WHO launched a program for the control of acute respiratory infections (ARI) with the main aim to reduce morbidity and mortality from ARI in children under-five years of age. Children identified with CAP were given antibiotics according to severity of disease [3, 4]. Also, immunization was identified as major strategy to prevent CAP. Therefore, in addition to vaccination against measles, vaccines against HiB and SP would also contribute to reduction of burden of CAP.

CAP contributes to 0.44 million deaths of under-five children in South East Asian region [5].Child Health Epidemiology Reference Group`s (CHERG) pneumonia working group published estimates on morbidity and mortality of clinical pneumonia for 192 countries including India [6]. Still, community-based estimates of incidence of CAP in under-five children from the specific Indian states and districts are lacking. Lack of reliable estimates of CAP from Indian states would hamper assessment of impact of various strategies for its prevention or treatment. Hence, the current prospective study was done to estimate the incidence of CAP in two districts each within the states of Uttar Pradesh and Bihar, India. These states have poor maternal and child health indicators [7].

## Materials and methods

### Study setting

The incidence of CAP was computed for two districts of Uttar Pradesh (Etawah and Lucknow) and two districts of Bihar (Patna and Darbhanga)in North India for time period of January to December, 2016. Uttar Pradesh is the most populous state in India and is divided into 75 administrative districts. Bihar is the third most populous state in India and is divided into 38 administrative districts. Infant mortality rate and under-five mortality rate in both states are higher than the national average (Table 1). Both states have immunization against measles and HiB in the universal immunization schedule [8, 9] but not against SP in the year 2016. According to a publication by WHO, in the year 2016, HiB vaccine coverage of Uttar Pradesh was 70%-79%and Bihar >90% [10].

**Table 1:** Socio-demographic and health indicators of India and study districts.

Indicators [Ref]	India	Uttar Pradesh		Bihar	
		Lucknow	Etawah	Patna	Darbhanga
Area (Sq. Km.)[11]	3287263	2528	2311	3202	2279
Population (million)[11]	1210.60	3.39	1.20	5.80	3.90
Density of Population [11]	382	1816	538	1823	1728
Urban Population (%)[11]	31.20	66.2	22.3	43.07	9.74
Children 2–59 Months (million) [11]	164.50	0.36	0.15	0.63	0.48
Literacy Rate (%) [11]	73	77.3	78.4	70.7	68.3
Crude Birth Rate [11] (births per 1,000 population)	19.27	18.2	21.8	21.1	26.2
Infant Mortality Rate (per 1,000 live births)	41[11]	44 [12]	56[12]	31[12]	44[12]
Under-5 Mortality Rate (per 1,000 live births)	50[12]	58 [12]	85 [12]	46 [12]	77[12]
Children under 5 years who are stunted (height-for-age) (%) [13]	38.40	53.2	37.5	43.5	49
Children under 5 years who are wasted (weight-for-height) (%) [13]	21.0	11.40	33.60	28.50	16.60
Children under 5 years who are severely wasted (weight-for-height)(%)[13]	7.50	2.30	17.90	12.10	4.90
Children under 5 years who are underweight (weight-for-age) (%)[13]	35.70	32.60	44.50	49.20	41.10
Measles Vaccination in children 12–23 months [13] (%)	81.10	79.90	66.90	84.20	75.40
Children age 12–23 months who received 3 doses of DPT vaccine (%) [13]	78.4	72.2	62.8	85.1	72.0
Children with fever or symptoms of ARI in the last 2 weeks preceding the survey taken to a health facility (%)[13]	73.2	78.7	66.9	64.5	54.6

The public health system in India is structured in three tiers viz. primary, secondary and tertiary providing community-based preventive services and patient care. The primary tier is in the rural areas and designed to have three types of health care facilities, namely a Sub-Centre for a population of 3000–5000, a Primary Health Centre (PHC) for population of 20000–30000 and a Community Health Centre (CHC) for population of 80,000–120,000.PHC is manned by a medical doctor supported by skilled staff. PHCs are linked to a CHC which is a 30-bedded hospital with outpatients, investigative and in-patient treatment facilities primarily for maternal and child care. It has a Block Medical Officer/Medical superintendent, a Public Health specialist, a Public Health Nurse and skilled support staff. In the second tier are sub-district and district hospitals located in urban areas established over a population of approximately 5,00,000. Tertiary care is provided by certain upgraded district hospitals and medical teaching and training academic institutions mostly located in urban areas.

In addition to the public health system there is large, less regulated private health care system in rural and urban areas of India. The private health care providers may be qualified or unqualified. Qualified health care providers practice modern medicine or alternative medicine (Ayurveda, Yoga and Naturopathy, Unani, Siddha and Homoeopathy) in urban and semi-urban areas. In the rural areas, there is predominance of health care seeking from unqualified health providers such as pharmacists or medical store keepers, faith healers and traditional healers which often results in delay in treatment and inadequate and improper dosing [14–16].There is also self-administration of drugs purchased over the counter. The private health care is delivered through small private clinics of solo practitioners or through small/large hospitals which are largely unregulated. Apart from these, there are not-for-profit/charitable hospitals or corporate hospitals again in large cities and in urban areas.

### Study methodology

#### Ethical approval

Ethical approval for community survey as well as hospital-based surveillance study was obtained from Health Ministry Steering Committee of Indian Council of Medical Research, New Delhi, India as well as the Institutional Ethics Committee of King George’s Medical University, Lucknow, India.

#### Community survey

A community survey of health care seeking behavior was conducted in rural Lucknow (Bill & Melinda Gates Foundation Grant No: OPP1084307). Lucknow district is divided into eight rural blocks and has 807 villages. Data on population and number of household per village was obtained from Census of India (2011–2012) [11].Using this data, villages were stratified as small (1–200 households), medium (201–400 households) and large-sized villages (≥401 households).Thereafter, based on 30 cluster-sampling methodology, villages were selected to have proportionate representation of small, medium and large sized villages [17]. Detailed methodology has been published elsewhere [18].

In each village, ten households with at least one child in age group 2–59 months were approached. If a house had more than one child of eligible age-group then all were included. For survey, a house with an eligible child was identified from approximate center of the village. Thereafter in each of the four directions from the centre of the village, two households with eligible child/children were selected, one in the periphery and the other between center and periphery. After obtaining written, informed consent from head of the household or parents of the eligible child, pre-tested and structured interview schedules were administered by trained interviewers. Socio-demographic information of the household was collected from the primary respondent (preferably the mother). Each respondent was asked “*if a child between 2–59 months had symptoms of cough along with fast breathing (with or without chest in-drawing)*” in the last 12 months and if any such child was hospitalized for these symptoms **[S1 Appendix]**.

#### Hospital-based CAP surveillance network

As a part of a separate study, a surveillance network of public and private hospitals that admitted children < 5 years was formed after obtaining their written, informed consent to participate(Bill & Melinda Gates Foundation Grant No: OPP1118005). Detailed methodology has been published elsewhere [19]. Children hospitalized in the network hospitals with history of fast breathing with or without chest in-drawing, were screened [3].Included were children aged 2–59 months, hospitalized with symptoms of WHO defined CAP [3]who resided in the study index district after informed, written parental consent. Excluded were those with cough and respiratory symptoms for more than 14 days (to exclude tuberculosis) [19]. Included children were followed up in the hospital to assess outcomes such as mortality.

For screening and recruiting children, surveillance officers were hired and trained. They did regular visits to these hospitals according to a set schedule. In between their visit, telephonic passive surveillance was done and the hospitals were also encouraged to telephonically contact the surveillance office when a suspected CAP was admitted. In these situations, unscheduled visits were made to such facilities for screening and recruitment [19]. On hospital visit, surveillance officers screen admission registration of network hospital for potential admissions of CAP. Cases that fulfilled inclusion criteria and had no exclusion criteria were enrolled. Socio-demographic, clinical anthropometric and laboratory/diagnostic tests information was collected from hospital records or by interviewing parents on a standardized and pre-tested questionnaire **[S2 Appendix].**

### Data management and statistical analysis

Double data entry of community survey was done in Microsoft Excel by two independent operators. Data of hospital surveillance network was entered online in customized software by all four participating sites. Secondary entry of hospital surveillance data was done in Lucknow site in different customized software. After data cleaning, analysis was done using SPSS version 18 (Chicago, IL) [20]. Exploratory data analysis was performed for outlier detection and missing observations. Descriptive statistics for continuous variables and frequency distribution for categorical variables were generated and compared for district wise variability. Categorical variables across district were compared using chi square test. A p value of <0·05 was taken as statistically significant using a two-tailed distribution. Weight-for-age of each child was calculated using EPI software [21] and Weight-for-age z≤3 was taken as underweight. The incidence of CAP for the year 2016 and its 95% confidence interval was computed using the methodology given below.

#### Methodology to estimate the incidence of CAP

Incidence of CAP in four study districts was estimated using data from the following three sources:

1. Admissions within the hospital surveillance networks of the four study districts
2. Proportion of CAP hospitalized for pneumonia from the cross-sectional community survey
3. Population of children under 5 years of age from Census of India (2011–2012)[11]

A mathematical model was developed to estimate the cases of CAP in the community in four study districts using the following seven steps.

1. *Child Population Data for establishing denominator*: Data has been taken from Census of India (2011–2012) for under 5 years of age. The census data was categorized into 2–11 months and 12–59 months age-groups in four study districts [11].
2. *Hospitalization Fraction (HF) of CAP*: HF for each age-category of CAP was obtained from the community survey.
  HF = Number of hospitalized cases of CAP /Total number of cases of CAP within the same time frame
3. *Total hospitalized cases of CAP within study district in the year 2016*: We counted all cases of CAP admitted in network hospitals. They were either from the study district or adjoining districts. We assumed that cases from study district would likewise be getting hospitalized for the treatment of CAP in hospitals of adjoining districts which were out of surveillance network. Hence, total cases of hospitalized CAP from study district were taken to be sum of cases from study district plus adjoining districts admitted in our hospital network. We observed that there was clustering of network hospitals in the centre of district. The average estimated distance from the city centre to the farthest boundary of a district was on an average 90 km which is equal to commuting time of about 2 hours on a four-wheeler by road. Hence, families in the peripheral areas are more likely to seek hospital care from adjoining district hospital closer to residence.
  We tested homogeneity between four study districts among proportion of cases hospitalized for CAP from same districts, proportion of female and proportion of underweight cases. For this we compared the difference among all pairs of proportions using Marascuilo procedure [22].
4. *Child Population Per Hospital (PPH)*:We calculated the average PPH for all four districts. It was calculated using the following formula
  PPH = (n of children of age category as per District Census 2011–2012) / (n of reporting hospitals)
5. *District Correction Factor (DCF)*: To overcome heterogeneity among proportion of children (point 4 above), DCF was calculated taking Lucknow as reference district. DCF was used for2-11months, 12–59 months and 2–59 months age categories.
  DCF was calculated as follows:
  DCF = PPH in Lucknow or Etawah or Patna or Darbhanga / PPH reported in Lucknow District
6. To estimate incidence of children under 5 years of age in Lucknow, Etawah, Patna, Darbhanga districts
  I _Lucknow / Etawah/Patna/Darbhanga =_ [{(total hospitalized cases in Lucknow, Etawah or Patna or Darbhanga in 2016)/HF}/(total population in the category by 2011–2012 Census of India)*DCF*1000]
7. Bootstrap method was used to estimate 95% confidence interval for the incidence of CAP per 1000 children.

## Results

The community survey was conducted in 240 villages of Lucknow district during February-May 2016. From 2400 households 3351 children in the age group 2–59 months were included in the study. Of these 25.70%were 2–11 months of age and 74.27% were 12–59 months of age. 24.58% (824/3351) children suffered from CAP in last 12 months and out of these 4% [95% CI: 3%-5%] (33/824) children (2–59 months) were hospitalized. Of these 4% [95% CI: 2%-6%](10/248)children were between 2–11 months of age and 3% [95% CI: 2%-5%](23/576) in age group 12–59 months (Table 2).

**Table 2:** Proportion of cases of possible pneumonia hospitalized in last 12 months in Lucknow district based on community survey (2016).

Age–group (months)	Surveyed (n)	Cases of possible Pneumonia (n)	Hospitalized for possible pneumonia (n)	Hospitalization Fraction (95%CI)
2–11	862	248	10	0.04(0.02–0.06)
12–59	2489	576	23	0.03(0.02–0.05)
2–59	3351	824	33	0.04(0.03–0.05)

In Hospital-based surveillance study, 5172 children were recruited from 117 hospitals from January to December, 2016. Number of hospitals in districts that admitted children were as follows: Lucknow (n = 50), Etawah (n = 15), Patna (n = 23) and Darbhanga (n = 29).We assumed that these children admitted in 117 hospitals represent 4% (95%CI: 3%-5%) of all incident cases for CAP. Table 3 describes the distribution of children hospitalized for CAP from study districts and adjoining districts in hospital-based surveillance study in year 2016. It was noticed that overall 40.06% (2072/5172) children were hospitalized in network hospitals from study district and 59.94% (3100/5172) were from adjoining districts and this was dissimilar across four sites (p < 0.0001). The proportion of females among recruited cases of CAP was 28.71% (1485/5172)(Table 3).We found that there was heterogeneity among proportion of female cases across the four sites (p < 0.0001).Hospital-based age-wise CAP case fatality in our study districts was 0.91% (13/1422) in 2–11 months and 0.46% (3/650) in 12–59 months age group.

**Table 3:** Distribution of hospitalized children for community acquired pneumonia from study district, adjoining districts and female children in hospital based surveillance study in year 2016.

Study Districts	Children from study district n (%)	Children from adjoining districts n (%)	Total children n (%)	Female children n (%)
Lucknow	505(40.86)	731(59.14)	1236	425(34.38)
Etawah	260(34.21)	500(65.79)	760	211(27.76)
Patna	658(38.23)	1063(61.77)	1721	476(27.65)
Darbhanga	649(44.60)	806(55.40)	1455	373(25.63)
Total	2072(40.06)	3100(59.94)	5172(100.0)	1485(28.71)
p value	<0.0001			<0.0001

Table 4 reports the number and proportion of underweight (weight-for-age z≤3) children between 2–59 months of age hospitalized for CAP in each district. The proportion of underweight children in four study districts was 12.59% (261/2072). It was found that there was dissimilar proportion of underweight children across four districts (p < 0.0001).

**Table 4:** Underweight (weight-for-age z≤3) children (2–59 months) hospitalized for community scquired pneumonia in each district.

Study Districts	Underweight Children n (%)	Total Children n (%)
Lucknow	81(16.03)	505
Etawah	37(14.23)	260
Patna	96(14.58)	658
Darbhanga	47(7.24)	649
Total	261(12.59)	2072(100.0)
p value	<0.0001	

In Table 5, the data which was used to compute DCF has been given. Using Lucknow district as reference, DCF was applied to all study districts-Etawah, Patna and Darbhanga.

**Table 5:** Computation of districts correction factor for various age categories based on census 2011–2012 and reporting hospitals in surveillance data.

Study District	Age–Group (Months)	Number of children as per district census (2011–2012)	Number of reporting Hospital	Population per hospital (PPH)	PPH Lucknow	District Correction Factor (DCF)
Lucknow	2–11	63028	50	1260.56	1260.56	1
	12–59	294187	50	5883.74	5883.74	1
	2–59	357215	50	7144.30	7144.30	1
Etawah	2–11	27745	15	1849.66	1260.56	1.47
	12–59	125747	15	8383.13	5883.74	1.42
	2–59	153492	15	10232.80	7144.30	1.43
Patna	2–11	97690	29	3368.62	1260.56	2.67
	12–59	531789	29	18337.55	5883.74	3.12
	2–59	629479	29	21706.17	7144.30	3.04
Darbhanga	2–11	82102	23	3569.65	1260.56	2.83
	12–59	406321	23	17666.13	5883.74	3.00
	2–59	488423	23	21235.78	7144.30	2.97

Table 6 reports the incidence of CAP per 1000 child-year of all study districts for the year 2016. It was found that in Uttar Pradesh state, Lucknow district had lower incidence of CAP as compared to Etawah district. We also found higher incidence of CAP in Darbhanga district as compared to Patna district in Bihar state.

**Table 6:** Estimation of the incidence of community acquired pneumonia for various age categories based on community survey and hospital based surveillance for four study districts.

Study Districts	Age–group (In Months)	Number of children in district (census 2011)	Total children hospitalized for CAP	Hospitalization fraction (HF) based on community Survey, Lucknow^¶^	Estimated Incidence cases of pneumonia	District Correction Factor DCF*	Incidence of CAP per 1000 child-year (95%CI)
Lucknow	2–11	63028	689	0.04	17225	1	273.20 (269.96–276.62)
	12–59	294187	547	0.03	18233	1	61.97 (61.19–62.76)
	2–59	357215	1236	0.04	30900	1	86.50 (85.72–87.29)
Etawah	2–11	27745	568	0.04	14200	1.47	752.34 (746.67–758.04)
	12–59	125747	192	0.03	6400	1.42	72.27 (71.10–73.45)
	2–59	153492	760	0.04	19000	1.43	177.01 (175.44–178.58)
Patna	2–11	97690	1251	0.04	31275	2.67	854.77 (851.85–857.73)
	12–59	531789	470	0.03	15667	3.12	91.916 (91.53–92.31)
	2–59	629479	1721	0.04	43025	3.04	207.78 (207.20–208.37)
Darbhanga	2–11	82102	1058	0.04	26450	2.83	911.71 (908.58–914.85)
	12–59	406321	397	0.03	13233	3.00	97.706 (97.12–98.29)
	2–59	488423	1455	0.04	36375	2.97	221.18 (220.40–221.97)

^**¶**^ Refer Table 2

*Refer Table 5

## Discussion

This study was done to estimate the incidence of CAP in children under-five years of age residing in two districts of Uttar Pradesh and two districts of Bihar, as there is scarcity of such data. We found that incidence of CAP per 1000 child-year for Lucknow District was86.50 (95% CI: 85.72–87.29); Etawah district 177.01(95% CI: 175.44–178.58); Patna district 207.78 (95% CI: 207.20–208.37) and Darbhanga district 221.18 (95% CI: 220.40–221.97). This incidence was calculated from an estimated 4% hospitalization rate of CAP obtained from the cross-sectional survey of rural Lucknow district in same year, admissions in hospital based surveillance network established in four study districts, and population data from Census of India (2011–2012) [11].

Various researchers have reported the incidence of CAP in children under-five years of age from South East Asian countries, including India, either by performing a systematic review or mathematical modeling [1, 5–6]. HiB vaccination was introduced in India in 2011 in a phased manner [8]. Prior to introduction of HiB vaccination, median incidence of CAP of 0.37per child-year for India was reported by Rudan et al [1], 0.28per child- year (95% CI 0.11–0.51)by Child Health Epidemiology Reference Group(CHERG)[6] and 0.26 per child-year (95% CI 0.13–0.61) for South East Asian region by Walker et al [5]. In addition, CHERG reported that 11.5% (95% CI 8.0–33.0%) of CAP cases had severe pneumonia [6], in contrast to 8.7% from an earlier study [1].

The current study was done post introduction of HiB vaccination in India. Lower range of incidence of CAP per 1000 child-year reported by us (86.50, 95% CI: 85.72–87.29) is understandably below the incidence of CAP reported in studies quoted earlier [1,5–6]. District-wise incidence found by us is in close approximation with region-wise incidence of lower respiratory infection **(**LRI) of 133.9 (95% UI 107–165)reported by the Global Burden of Disease (GBD) study, 2016 [23]. GBD researchers combined incident cases of bronchiolitis and pneumonia and reported them as LRI. In the current study as well as in studies done by others [1, 24–25], WHO definition of CAP is used. WHO definition of CAP includes both the clinical entities of bronchiolitis and pneumonia [3] and hence can be interchangeably used with LRI.

Gupta and colleagues [25] have reported incidence of clinically severe pneumonia requiring hospitalization while conducting the HiB vaccine trial in four sites in India. The incidence of severe pneumonia requiring hospitalization varied from 27.20 per 1000 child-year in Chandigarh city to 78.9 per 1000 child-year in Kolkata for year 2005–2007. Farooqui et al. using mathematical modeling also reported incidence of severe pneumonia to be 41.4 (95% CI 38.0–45.2) per 1000 child-year and 45.5 (95% CI 41.7–49.6) per 1000child-year in the states of Uttar Pradesh and Bihar, respectively [24] for the same time period. Hence there is variability in the incidence of severe CAP by city as well as district.

We have found that children in the age category of 2–11 months had 5to10times higher incidence of CAP than those in 12–59 months age category. Similar findings were reported by CHERG study group [6] who found 3 times higher in incidence of severe pneumonia in children aged 2–11 months. Similarly, Gupta et al. also reported that severe CAP is much more common in younger age group (<1yr) compared to older children (1–2 years) [25].

In our community survey we have found that 4% children with clinical features of CAP were hospitalized. We assume that those hospitalized were likely to be suffering from severe pneumonia. This is because cases of severe pneumonia develop danger signs which alarm caregivers who then take them to hospital. Since the proportion of severe pneumonia among those who get CAP varies from 11.5% to 8.7% [1, 5], it means that approximately half or more cases of severe pneumonia in our Indian study districts still do not get adequate care, which is alarming. This is likely to be the reason for high burden of deaths due to CAP in the two states of Uttar Pradesh and Bihar as well as in India [2]. Hence there is an urgent need to reduce the incidence of severe CAP with introduction of preventive measures such as introduction of conjugate vaccine against SP (PCV) [26].

We have used concurrent data of cross sectional community survey to find the proportion of clinical pneumonia cases who were hospitalized. Another strength of this study is that we have established a surveillance of network hospitals in four districts and have prospectively accrued all eligible hospitalized pneumonia cases. We have used a standard WHO definition for pneumonia [3] in community survey and in hospital based surveillance. This has given our study internal and external validity and is generalizable.

However, the cross-sectional community survey was done only in rural areas of Lucknow district and has not been replicated in other three study districts. Proportion of cases of CAP hospitalized in other districts may not be same as in Lucknow as these three districts that have poorer health infrastructure [7]. This is a limitation. To overcome this, we have calculated and applied DCF which takes into account the number of children under 5 years of age per available hospital beds. This is justified by the fact that while rest of India has hospital bed to population ratio of1:1833, Bihar has 1:8800 which is 4.8 times lower [27].Our conservative estimate of DCF for Bihar is approximately3.0 (Table 5). Unlike another modeling exercises for incidence of pneumonia [6,24], we have not done any corrections for nutritional status, low birth weight and use of solid–fuel as we worked on real time data. This makes data pragmatic.

In our study, among 5172 hospitalized cases of CAP, 28.7% (1485/5172) were females. This can be attributed to gender bias against females in health care seeking in India as has been reported previously by other researchers [28–30]. We also documented that only 4% cases of CAP get hospitalized, hence there is a possibility of an uncounted proportion of cases dying prior to hospitalization. Both these issues could have lead to underestimating the true incidence of CAP.

We have found that there is a district-wide variation in the incidence of CAP. This may be partly explained by poor health infrastructure in Bihar [7] and variable care-seeking for acute respiratory infections (ARI)(Table 1). However further community based research is needed to ascertain causes of district to district variations in incidence of CAP.

### Conclusion

Incidence of CAP in Uttar Pradesh and Bihar is high, being much higher in infants. Hence there is an urgent need for introduction of preventive strategies, improving health seeking behavior and quality of care for CAP.

## Supporting information

S1 Appendix (PDF)

S2 Appendix (PDF)
