# Supplementary material for: Incidence of community acquired pneumonia in children aged 2-59 months of age in Uttar Pradesh and Bihar, India, in 2016: An indirect estimation
Source: PLoS One. 2019 Mar 20;14(3):e0214086. doi: 10.1371/journal.pone.0214086 (PMC6426182; doi:10.1371/journal.pone.0214086)
Supplement: S1 Appendix — (PDF) [file pone.0214086.s001.pdf]

ID No.: [ ] / [ ] [ ] [ ] [ ] [ ] [ ]/ [ ] [ ] [ ] [ ]
Block/ Village code / Household Code

Community Survey

"To Assess the Effectiveness of Various Communication Strategies for Improving Childhood Pneumonia Case Management: A Community Based Behavioral Open Labeled Trial in Rural Lucknow, Uttar Pradesh, India"

		[ ] Child Number	
1	Name of Child		
22	Age: (in completed months)	[ ] [ ]	
2	Date of Birth: DD/MM/YYYY	_ _ /_ _ /_ _ _ _	
23	Gender: (Code: 1-Male, 2-Female)	[ ]	

3.A	**a.** Symptom	**b.** Did this child suffer from this symptom in last twelve months?	**c.** What type/s of treatment was given to the child? **(Multiple response)** (Code: 1-No Treatment 2-Home remedies 3-Medicines from medical store 4-Medicines prescribed by doctor)	**d.** Who was the **first** healthcare provider whom you approached for treatment? (Code: 1 - Private Doctor 2-ANM 3Government Hospital 4-Others)	**e.** What was the qualification of the Doctor? (Code: 1-Not Known 2-Unqualified 3-M.B.B.S. 4-M.D. 5-Ayush)	**f.** Apart from thefirst healthcare provider, where else did you seek Medical Care? **(Multiple response)** (Code: 1-ANM 2- PHC 3-CHC 4-District Hospital 5-Private Doctor 6-Private Hospital 7-Did not visit any other place)	**g.** From where all/whom did you get medicine? **(Multiple response)** (Code: 1-From ANM 2- From PHC 3-From CHC 4-Private Doctor 5-Private Hospital 6-Any other, specify)	**h.** Did you get any instructions along with medicines? (Code: 1-Yes, 2-No)	**i.** If yes, instructions were given in which form? **(Multiple response)** (Code: 1-Oral 2- Hand written 3-Card)	**j.** Were you advised follow-up care after 72 hours?	**k.** If yes, did you go for the follow-up after 72 hours?
3.B	Running nose	Yes [ ] No [ ]	[ ] [ ] [ ]	[ ]	[ ]	[ ] [ ] [ ]	[ ] [ ] [ ]	[ ]	[ ] [ ]	Yes [ ] No [ ]	Yes [ ] No [ ]
3.C	Cough with fast breathing (with or without chest in drawing)	Yes [ ] No [ ]	[ ] [ ] [ ]	[ ]	[ ]	[ ] [ ] [ ]	[ ] [ ] [ ]	[ ]	[ ] [ ]	Yes [ ] No [ ]	Yes [ ] No [ ]

4	Was the child admitted in hospital, for treatment of pneumonia, in last 12 months?(Code: 1-Yes, 2-No)	[ ]
5	In which type of hospital, was the child was admitted **first**? (Code: 1 – Government Hospital, 2 – Private Hospital)	[ ]
6	Why did you prefer this hospital? **(Multiple response)** (Code: 1-Near to my house, 2-Better Doctors, 3-Better facilities, 4-Round the clock service, 5-Affordable treatment, 6-Other,specify……………………)	[ ] [ ] [ ] [ ] [ ] [ ]
7	Was the child referred/admitted to other hospital/s also? (Code: 1-Yes, 2-No)	[ ]
8	What type/s of hospital/s was/were they? **(Multiple response)** (Code: 1 – Government Hospital, 2 – Private Hospital)	[ ] [ ]
**Note: If treatment records are available, please collect a copy.**		
9	Name of Interviewer	
10	Signature of Interviewer	
11	Date of Interview	[ ] [ ]/[ ] [ ]/[ ] [ ] [ ] [ ]
