# Supplementary material for: Incidence of community acquired pneumonia in children aged 2-59 months of age in Uttar Pradesh and Bihar, India, in 2016: An indirect estimation
Source: PLoS One. 2019 Mar 20;14(3):e0214086. doi: 10.1371/journal.pone.0214086 (PMC6426182; doi:10.1371/journal.pone.0214086)
Supplement: S2 Appendix — (PDF) [file pone.0214086.s002.pdf]

District Childhood Pneumonia Surveillance

Department of Paediatrics, King George's Medical University, Lucknow

Clinical Data Form (Form C)

(Instructions- Fill data in all fields; if data not available write 9/99/999 as per field length NI=No Information)

1. ID No.: [__] / [__] / [__][__][__]/[__][__][__][__] 2. Contact No: [__][__][__][__][__][__][__][__][__][__][__][__]
State/District/ Unit / Subject number (*For office use*)

3. Date of admission: [__][__]/[__][__]/[__][__][__][__] d d / m m / y y y y
4. Time (*tick one*): Day ☐ / Night ☐

5a. Age in months: [__][__]
5b. Date of Birth: [__][__]/[__][__]/[__][__][__][__] d d / m m / y y y y

6. Gender (*tick one):* Male ☐ / Female ☐

7. Place of Residence (*tick one*): Rural ☐ / Urban ☐
8. Family Type (*tick one*): Joint ☐ / Nuclear ☐ /NI ☐

9. Mother's age *(in years)* [__][__]

10. Mother's education [__]
11. Father's education [__]
(1=Uneducated, 2=No formal education, 3= Class I to V, 4= Class VI to X, 5= Class XI-XII, 6= Graduate, 7=Post Graduate)

12. Number of living siblings: [__][__]
13. Birth Order: [__][__]

14. Length/Height (*in cm):* [__][__][__].[__]
15. Weight *in Kilograms* [__][__].[__]

16. Upper mid-arm circumference *(in cm):* [__][__].[__]
17. Head circumference *(in cm):* [__][__].[__]

18. Immunization status *(tick one):* Complete for Age ☐ Incomplete for Age ☐ Unimmunized ☐ Don't Know ☐

18.1 Has the child been given pneumococcal Conjugate Vaccine (PCV)?
1. Yes [ ] 2. No [ ] (go to Q. 19) 3. NI [ ] (go to Q. 19)

18.2 if yes, please tell when it was given

**Dose**	**Date**	**Month**	**Year**
PCV Dose-1 (recommended at 6 weeks of age)	[ ][ ]/	[ ][ ]/	[ ][ ] [ ][ ]
PCV Dose-2 (recommended at 14 weeks of age)	[ ][ ]/	[ ][ ]/	[ ][ ] [ ][ ]
PCV Dose-3 (recommended at 9-15 months of age)	[ ][ ]/	[ ][ ]/	[ ][ ] [ ][ ]

19. Ever Breastfed (*Tick one)*: Yes ☐ No ☐ NI ☐

20. Currently Breastfeeding (*Tick one)*: Yes ☐ No ☐ NI ☐
21. Breast Feeding duration in months [__][__]

22. Father's Occupation: [__]
23. Mother's Occupation: [__]
(1=Unemployed/Homemaker 2=Daily wages 3=Salaried 4=Business 5=Farmer 6=Professional 7=No Information)

24. Father Smoker (*Tick one*): Yes ☐ No ☐ NI ☐
25. Father Indoor smoker (*Tick one*): Yes ☐ No ☐ NI ☐

26. Mother Smoker (*Tick one*): Yes ☐ No ☐ NI ☐
27. Mother Indoor smoker (*Tick one*): Yes ☐ No ☐ NI ☐

28. Any other smoker in family (*Tick one)*: Yes ☐ No ☐ NI ☐

29. Family Member Indoor smoker (Tick one): Yes ☐ No ☐ NI ☐

30. Type of house (*Tick one)* : Kuchha ☐ Pucca ☐ Semi- constructed ☐

31. Number of rooms in the house: [__][__]
32. Separate cooking space (*Tick one)*: Yes ☐ No ☐

33. Fuel for cooking (*Tick all applicable)*: LPG ☐ Kerosene ☐ Coal ☐ Dung Cake ☐ Electricity ☐ Wood ☐

Episodes of similar illness in the past

34. History of fast breathing & cough (Tick one): Yes ☐ No ☐
35. If 'Yes' Number of times in last 6 months [__][__]

History of presenting symptoms with duration on admission

Symptoms on admission	Findings (tick one)	If Yes, duration in Days/Hours
36. Fever	Yes ☐ No ☐ NI ☐	[__][__]/[__][__]
37. Cough	Yes ☐ No ☐ NI ☐	[__][__]/[__][__]
38. Fast breathing	Yes ☐ No ☐ NI ☐	[__][__]/[__][__]
39. Difficult Breathing	Yes ☐ No ☐ NI ☐	[__][__]/[__][__]
40. Inability to Drink	Yes ☐ No ☐ NI ☐	[__][__]/[__][__]
41. Vomiting	Yes ☐ No ☐ NI ☐	[__][__]/[__][__]
42. Blue discolouration of lips	Yes ☐ No ☐ NI ☐	[__][__]/[__][__]
43. Convulsions	Yes ☐ No ☐ NI ☐	[__][__]/[__][__]
44. Lethargic/Unconscious	Yes ☐ No ☐ NI ☐	[__][__]/[__][__]

45. **Clinical Examination Date:** [__][__]/[__][__]/[__][__][__][__] 46.**Time (hr/min)**[__][__] / [__][__]

P.T.O.

Examination findings at admission (Abstracted from Case Sheet)	Findings
47. Respiratory rate (count for 1 minute)	[__][__][__]
48. Heart rate (count for 1 minute)	[__][__][__]
49. Pulse Pressure (*Tick one)*	Normal ☐ Low ☐ NI ☐
50. Fever (*Tick one)*	Present ☐ Absent ☐ NI ☐
51. Conjunctival Pallor (*Tick one)*	Yes ☐ No ☐ NI ☐
52. Central Cyanosis (*Tick one)*	Yes ☐ No ☐ NI ☐
53. Peripheral Cyanosis (*Tick one)*	Yes ☐ No ☐ NI ☐
54. Breathing difficulty (*Tick one)*	Yes ☐ No ☐ NI ☐
55. Noisy Breathing at admission (*Tick one)*	Yes ☐ No ☐ NI ☐
56. Pulse Oximetry Oxygen Saturation	Yes ☐ No ☐ NI ☐<br>(if yes) Oxygen Saturation in % [__][__][__]%
57. Pulse Oximetry with O_2/without O_2	With O_2 ☐ Without O_2 ☐ Not sure ☐
58. Breath Sound on auscultation *(tick one or both)*	Vesicular ☐ Bronchial ☐ NI ☐
59. Breath Sounds (Intensity) *(tick one or both)*	Normal ☐ Diminished ☐ NI ☐
60. Crepts on auscultation*(tick one)*	Yes ☐ No ☐ NI ☐
61. Rhonchi/Wheeze on auscultation*(tick one)*	Yes ☐ No ☐ NI ☐
62. Cardio-Vascular System	Normal ☐ Abnormal ☐ Specify......................................

Investigations: X-ray Chest PA view 63. Type of X-ray *(tick one):* Digital ☐ Plain ☐ No X-ray ☐

64. X-ray Date: [__][__]/[__][__]/[__][__][__][__] **(*send report 1*)**

65. Urine Sample collected *(tick one):* Yes ☐ No ☐66.Urine Sample Collection Date: [__][__]/[__][__]/[__][__][__][__]

**Lab Investigations *(First reports upon admission-send report 2*)**		
67. Haemoglobin *(tick one)*	Yes ☐ No ☐	Haemoglobin Value [__][__].[__]gm %
68. Total Leukocyte Count *(tick one)*	Yes ☐ No ☐	TLC value[__][__][__][__][__] cells per cubic mm
69. Differential Leukocyte Count *(tick one)*	Yes ☐ No ☐	N[__][__]% L[__][__] % E[__][__] % M[__][__]%
70. Platelets *(tick one)*	Yes ☐ No ☐	Platelets count[__][__][__][__][__][__][__]cells<br>(*tick one*) Adequate ☐ Inadequate ☐
71. C-Reactive Protein *(tick one)***(*send report 3*)**	Yes ☐ No ☐	CRP value [__][__][__].[__][__]mg/L OR<br>Positive ☐ Negative☐

Bacterial Culture *(If done, send report 4 & 5)*

72. Blood *(tick one)*<br>Yes ☐ No ☐ (Report 4)	Code for blood culture: [__],[__],[__]<br>if Any Other, Specify...	Code for Bacterial /Pleural fluid Culture :<br>**1** - S. pneumoniae, **2**- H. influenza,<br>**3** - S. aureus, **4**- MRSA,<br>**5** - Klebsiella sp., **6**- E. coli,<br>**7**- Pseudomonas sp., **8**- Acinetobacter sp.<br>9- Any Other
73. Pleural fluid *(tick one)*<br>Yes☐ No☐ (Report 5)	Code for Pleural fluid culture [__],[__],[__]<br>if Any Other, Specify......................................	

Pleural Fluid Examination (*If done, send-report -6)*

74. Protein *(tick one)* Yes ☐ No ☐	Protein (mg%) [__][__][__] . [__]
75. Cells *(tick one)* Yes ☐ No ☐	Cell count [__][__][__][__]<br>Cell type N[__][__]% L[__][__]% E[__][__]% M[__][__]%
76. Sugar *(tick one)* Yes ☐ No ☐	Sugar (mg%) [__][__][__]
77. Gram Stain *(tick one)* Yes ☐ No ☐	Positive ☐ Negative ☐
78. **Diagnosis by Physician:**	
79. **Final Outcome:** (*tick one)* Death ☐ Discharged ☐ LAMA ☐ Referred ☐	80. *If Referred, where* ....
**For office use:** 81. *Tick all applicable* (Report 1☐ 2☐ 3☐ 4☐ 5☐ 6☐) 82. Report uploading date: [__][__]/[__][__]/[__][__]	

83. Date of Data collection<br>[__][__]/[__][__]/[__][__][__][__]<br>84. Name of Data collector......................<br>85. Signature of Data Collector......................	86. Date of Data Verification<br>[__][__]/[__][__]/[__][__][__][__]<br>87. Name of Supervisor............................<br>88. Signature of supervisor............................	89. Date of Data entry<br>[__][__]/[__][__]/[__][__][__][__]<br>90. Name of DEO. ....<br>91. Signature of DEO..
92. Date of Secondary Data entry<br>[__][__]/[__][__]/[__][__][__][__]	93. Name of DEO	94. Signature of DEO ....

Signature of Principal Investigator............................
